# Prognostic Factors for Immune Thrombocytopenic Purpura Remission after Laparoscopic Splenectomy: A Cohort Study

**DOI:** 10.3390/medicina55040112

**Published:** 2019-04-18

**Authors:** Anna Kwiatkowska, Dorota Radkowiak, Michał Wysocki, Grzegorz Torbicz, Natalia Gajewska, Anna Lasek, Jan Kulawik, Andrzej Budzyński, Michał Pędziwiatr

**Affiliations:** 1 2nd Department of General Surgery, Jagiellonian University Medical College, Kopernika 21, 31-501 Kraków, Poland; anna.zychowicz@onet.pl (A.K.); dradkowiak@gmail.com (D.R.); m.wysocki@doctoral.uj.edu.pl (M.W.); grzegorz.torbicz@gmail.com (G.T.); natgajewska@gmail.com (N.G.); aniad303@gmail.com (A.L.); jankula@poczta.onet.pl (J.K.); andrzej.budzynski@uj.edu.pl (A.B.); 2 Centre for Research, Training and Innovation in Surgery (CERTAIN Surgery), 31-501 Kraków, Poland

**Keywords:** splenectomy, ITP, immune thrombocytopenia, laparoscopy, remission, long-term outcomes

## Abstract

*Background and Objectives:* Laparoscopic splenectomy (LS) has become the gold standard for patients with immune thrombocytopenic purpura (ITP). The total remission rate after splenectomy is 70%–90%, of which 66% is long-term. Despite this high response rate, some patients do not benefit from surgery. It is therefore important to try to identify risk factors for an unsatisfactory clinical response. The aim of this study was to assess long-term outcomes of LS for ITP and identify factors associated with increased disease remission rates. *Materials and Methods:* We retrospectively studied consecutive patients with ITP undergoing LS in a tertiary referral surgical center prospectively recorded in a database. Inclusion criteria were: Elective, laparoscopic splenectomy for diagnosed ITP, and complete follow-up. The cohort was divided into two groups—Group 1 (G1) patients with ITP remission after splenectomy and Group 2 (G2) patients without remission. There were 113 G1 patients and 52 G2 patients. Median follow-up was 9.5 (IQR: 5–15) years. *Results:* In univariate analysis, patient’s age, body mass index (BMI), preoperative platelet count, the need for platelet transfusions, and presence of hemorrhagic diathesis were shown to be statistically significant factors. Next, we built a multivariate logistic regression model using factors significant in univariate analysis. Age <41 years (odds ratio (OR) 4.49; 95% CI: 1.66–12.09), BMI < 24.3 kg/m^2^ (OR: 4.67; 95% CI: 1.44–15.16), and preoperative platelet count ≥97 × 10^3^/mm^3^ (OR: 3.50; 95% CI: 1.30–9.47) were shown to be independent prognostic factors for ITP remission after LS. *Conclusions:* The independent prognostic factors for ITP remission after LS revealed in our study are: age <41 years, BMI < 2 4.3 kg/m^2^, and preoperative platelet count ≥97 × 10^3^/mm^3^. Duration of the ITP and the time of treatment are not related to remission after LS.

## 1. Introduction

Immune thrombocytopenic purpura (ITP) is a hematological disorder characterized by autoimmune-mediated destruction of platelets and reduction of platelet production. In general, the mainstays of medical therapy are corticosteroids and intravenous immunoglobulins. However, long-term remission rates are only 20% to 25% in adults [1,2]. Splenectomy is a main second-line treatment in refractory ITP [3,4]. Since the very first laparoscopic splenectomy was carried out by Delaitre and Maignien in 1991, it has become the gold standard for patients with ITP [5]. The total remission rate after splenectomy is 70%–90%, 66% of whom have long-term remission without additional therapy [6]. Although the choice of surgical approach (laparoscopic) in case of splenectomy is straightforward due to the obvious benefits of minimally invasive surgery, the decision about splenectomy timing itself is still difficult [7,8,9]. The reason is that despite the high response rate of splenectomy, some patients do not benefit from surgery. For this reason, it seems reasonable to identify risk factors for satisfactory clinical response and failure in patients operated on for ITP. The aim of the study was to assess long-term outcomes of laparoscopic splenectomy (LS) for ITP and identify factors associated with increased remission rates.

## 2. Materials and Methods

### 2.1. Design

This was a retrospective cohort study of consecutive patients with ITP undergoing LS between 1998 and 2017 in a tertiary referral, university-affiliated, surgical center, that were prospectively recorded in a database. Inclusion criteria were: Elective, laparoscopic splenectomy for diagnosed ITP, and complete follow-up. Patients with unclear diagnosis of ITP, splenic trauma, initially submitted to open surgery, partial resections and other spleen-preserving procedures were excluded from further analyses. The entire cohort was divided into two groups—Group 1 patients with ITP remission after splenectomy and Group 2 patients without remission. All patients were followed up strictly for the first postoperative month. To collect long-term data, we contacted the patients and checked their remission status. The minimal time interval between surgery and follow-up was one year after surgery.

### 2.2. Definitions

Symptomatic ITP is defined as thrombocytopenia associated with petechiae or purpura, unusual/non-occasional hematomas, persistent bleeding from wounds or other injuries, mucosal bleeding, frequent or heavy epistaxis, and/or hemorrhage from any site (usually gingival or menorrhagia in women).

Remission (response) was defined as a platelet count of >100 × 10^3^/mm^3^ in follow-up, no symptoms of ITP and bleedings at the time of follow-up, and no current or postoperative treatment of ITP. Non-remission (non-response) was defined as a lack of a rise in platelet count to 100 × 10^3^/mm^3^ or an initial rise but return to values <100 × 10^3^/mm^3^ postoperatively. The need to restart or continue steroids or other therapy to sustain normal platelet count or spontaneous bleeding within 30 days after splenectomy was also considered non-remission (non-response). Additionally, we performed analyses for durable postoperative platelet count of >50 × 10^3^/mm^3^ in follow-up.

All patients were preoperatively assessed by a hematologist and appropriate treatment introduced according to our predefined protocol [10]. Operative time measurement was measured from skin incision to closure. The intraoperative blood loss was the amount of blood aspirated by the suction machine. Intraoperative adverse events were defined as any iatrogenic harmful event occurring during the operation not derived from the standard course of operation. Intraoperative blood loss ≥500 mL was considered to be hemorrhage, because losing less than one unit of blood (500 mL) usually does not negatively affect the condition of the patient and does not lead to hypovolemia and hemorrhagic shock [11,12]. Perioperative morbidity was defined as any complication or deviation from a routine postoperative course observed during 30 days after LS (graded with Clavien–Dindo classification [13]). Postoperatively, patients were followed-up by a hematologist or family doctor.

### 2.3. Operative Technique

The primary choice in all cases was four-port laparoscopic splenectomy. In the beginning of our experience in LS, we predominantly used the “vessels first” technique, as described elsewhere [14,15]. Later, the “hilar transection” technique was used, as we have described previously [16]. Occasionally, the operator placed clips on larger vessel branches of the hilum to ensure proper hemostasis. 

### 2.4. Ethics

This study meets the Report of the ISPOR Task Force on Retrospective Databases guidelines [17]. All procedures followed the ethical standards on human experimentation, both institutional and national, including the Fortaleza revision of the 1975 Declaration of Helsinki. The study was approved by the Bioethical Committee of the Jagiellonian University, Krakow, Poland (approval number 1072/6120/160/2017 from 21 December 2017). Informed consent for the surgical treatment was obtained from all patients before the procedure.

### 2.5. Statistical Analysis

Data analyses were performed using Statsoft STATISTICA v.13.5 software (Statsoft Inc., Tulsa, OK, USA). Continuous variables are presented as mean ± standard deviation (SD) or median and interquartile range (IQR), when appropriate. Categorical variables were analyzed in Pearson’s chi-square test or chi-square with Yates correction, when appropriate. The Shapiro–Wilk test was used to analyze if continuous variables were normally distributed. Continuous data was analyzed with the Student’s *t*-test (for normally distributed) and the Mann–Whitney U test (for non-normally distributed data). Finally, univariate and then multivariate logistic regression analyses were built in search of factors influencing odds ratios (OR) with 95% confidence interval (95% CI) of primary and secondary outcomes. Receiver operating characteristic (ROC) curves were used to set cut-off points in the process of conversion from a continuous to a dichotomous variable. A Kaplan–Meier curve was built for remission status within the study period. Results were considered statistically significant when the *p*-value was less than 0.05.

## 3. Results

### Patients

A total of 165 patients with ITP fulfilling inclusion criteria were treated in our department between 1998 and 2017. Study groups consisted of 113 patients with ITP remission (Group 1) and 52 without remission (Group 2). Baseline characteristics of the study cohort are presented in Table 1. Median follow-up was 9.5 (IQR: 5–15) years. There were no differences in gender between the groups. Patients in group 1 were significantly younger (*p* < 0.001), had a lower BMI (*p* = 0.002), and had a higher preoperative platelet amount (*p* = 0.034).

Complications occurred in 13 patients (7.88%), with nine (7.96%) of them in Group 1 and four (7.69%) in Group 2 (Table 2). There were no conversions. One patient died after surgery due to a pulmonary embolism, hence they were not observed long-term. The median hospital stay was four days (IQR 3–4), three days for Group 1 (IQR 3–4), and four days for Group 2 (IQR 3–5). 

**Figure 1:**
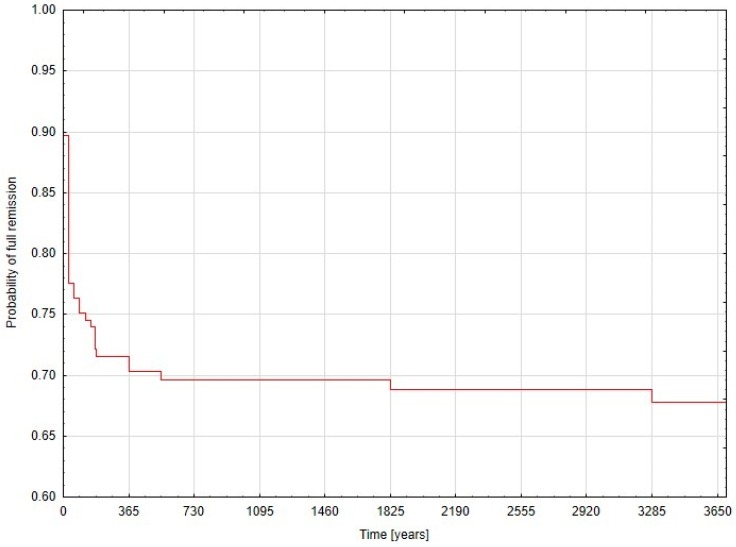
Probability of full remission against time of observation.

The Kaplan–Meier curve demonstrating probability of full remission against time of observation is presented in Figure 1.

Univariate logistic regression identified factors that may impact remission after LS (Table 3). In univariate analysis, age, BMI, preoperative platelet count, and the need for platelet transfusions were found to be statistically significant. Next, we built multivariate logistic regression model using items significant in univariate analysis (Table 4). Continuous variables used in univariate logistic regression models were dichotomized using ROC analyses. Cut-off points are shown in Table 4. Area under curve (AUC) for age was 0.72 (95% CI 0.63–0.81, *p* < 0.001), for BMI 0.71 (95% CI 0.62–0.80, *p* < 0.001), and for preoperative platelet count 0.61 (95% CI 0.51–0.70, *p* = 0.030). Age <41 years, BMI < 24.3 kg/m^2^, and preoperative platelet count ≥97 × 10^3^/mm^3^ were shown to be independent prognostic factors for ITP remission after LS.

Additionally, we performed logistic regression for possible risk factors for durable postoperative platelet count >50 × 10^3^/mm^3^. Table 5 shows the results of univariate logistic regression models. Age, preoperative platelet count, and preoperative platelet transfusions were found to be significant factors in univariate models. ROC analyses were built to set cut-off points for continuous variables. AUC for patient’s age was 0.74 (95% CI 0.64–0.84, *p* < 0.001), while for preoperative platelet count it was 0.55 (95% CI 0.41–0.69, *p* = 0.047). Then they were used in multivariate logistic regression, as presented in Table 6. Age <42 years and a preoperative platelet count ≥95 × 10^3^/mm^3^ were found to independently increase the odds ratio for durable postoperative platelet count above 50 × 10^3^/mm^3^. 

## 4. Discussion

In this study we evaluated factors associated with the long-term remission of ITP after LS to help surgeons to identify patients who are expected to benefit from splenectomy. Our study showed that an age <41 years, a BMI < 2 4.3 kg/m^2^ and a preoperative platelet count ≥97 × 10^3^/mm^3^ are independent factors increasing chance for remission. Regarding durable postoperative platelets at a level of >50 × 10^3^/mm^3^, an age <42 years and a preoperative platelet count ≥95 × 10^3^/mm^3^ were found to independently increase odds ratios.

Laparoscopic splenectomy is a safe procedure with low mortality and morbidity rates but is not completely free of complications. Asplenic patients are susceptible to opportunistic infections such as life-threatening, overwhelming post-splenectomy infections (OPSI), which is why all patients should receive preoperative vaccines for encapsulated bacteria [18]. According to American Society of Hematologist (ASH) 2011 guidelines, pneumococcal and meningococcal vaccinations for elective splenectomy are recommended and one dose of *H. influenzae* type b vaccine is not contraindicated before splenectomy [19]. A particularly important factor is the increased risk of venous thromboembolic events. In a study by Tastaldi et al. the perioperative morbidity was 7.3%, including three deep vein and two portal vein thromboses, one reoperation for bleeding, and no mortalities [20]. In a study by Thai et al. ITP patients who underwent splenectomy had a 16% venous thromboembolism rate when compared to a 2% rate on a matched cohort that was not treated with surgery [21]. Other studies have shown that the risk for portal or splenic vein thrombosis after splenectomy was estimated to be 0.1–4/100 patient-years [22]. Complications, as described in a work by Rijcken et al. occurred in 6/73 patients (8.2%), which consisted mostly of postoperative bleeding. Five patients (6.8%) required surgery for bleeding control. One patient had an epifascial wound infection after conversion. A limited pleural effusion developed in one patient [6]. In our study, complications occurred in 13 (7.88%) patients. Nine (7.96%) of them occurred in the remission group and four (7.69%) in the non-remission group. There were no conversions. One patient died after surgery due to pulmonary embolism. Although morbidity rates were relatively low, prediction of the efficacy of LS for ITP is still important.

There is no doubt that laparoscopic splenectomy is a valuable and effective treatment of patients with ITP but there are still patients who have a poor long-term response. The present study showed that two-thirds of ITP patients achieved a long-term response after LS during our follow-up periods and one third had no response. In a study by Tastaldi et al. after a median 62-month follow-up, two-thirds (68%) of the patients experienced a sustained response and were treatment-free [20]. Rijcken et al. found stable remission in a total of 44/72 (61.1%) patients [6]. Rui et al. showed that a total of 65 of 78 (83.3%) patients had stable remission and no need for further therapy for ITP after LS [23]. Differences in hematological outcomes might come from different definitions and clinical criteria used in different studies. We defined the response to LS based on recent consensus criteria for complete response (CR) as a platelet count of >100 × 10^3^/mm^3^ in follow-up [19]. However, it should be noted that studies like Istl et al. defined response to LS as a platelet count greater than 50 × 10^3^/mm^3^ with no need for postoperative medical management during the follow-up period [24], so we also performed analyses for that cut-off point. Age and preoperative platelet count remained significant, independent prognostic factors.

Many studies have attempted to indicate conclusive predictive factors of hematological response to splenectomy. Patient age is the most acknowledged predictive factor of successful outcome after splenectomy. The analysis of Duperier et al. revealed that a younger age predicted a successful response to laparoscopic splenectomy specifically in patients younger than 50 years. Conversely, refractory as well as recurrent disease were seen in older patients [25]. Duperier et al. also revealed that an age younger than 50 years had a sensitivity of 72%, a specificity of 68%, a positive predictive value of 58%, and a negative predictive value of 77% [25]. Tastaldi et al. showed that patients who sustained a long-term response were younger (44.7 years ± 20.6 vs. 53.4 years ± 19.5, *p* = 0.037) [20]. Other investigators Radaelli et al. [26] and Ojima et al. [3] have not found correlation between age and successful response to splenectomy. 

Preoperative platelet level has been evaluated as a predictive factor by many researchers. Rijcken et al. reported that patients with a high preoperative platelet count boosted with steroids and immunoglobulins had a stable long-term response [6]. Other studies presented similar results [20,23]. Duperier et al. revealed in a clinical study with a mean follow-up of 22 months that higher preoperative platelet levels predicted a successful response to LS regardless of how this level was achieved [25]. In our study the preoperative platelet count ≥97 × 10^3^/mm^3^ is a prognostic factor for ITP remission. On the other hand Ojima et al. showed that only a high platelet count on postoperative day seven was associated with a good response to splenectomy [3]. It is worth considering that maybe patients with initially higher platelet levels or those who respond to steroid therapy have less severe ITP. Other factors reported as successful predictors examined by some of investigators are disease duration from diagnosis to splenectomy, and splenic sequestration [27].

Timing of LS is not standardized. Most patients submitted to surgery have already failed in several medical attempts. They probably already have adverse effects such as hypertension, osteoporosis or infections [6]. In our study we observed that duration of ITP did not affect response to surgery. There is however one important factor that has not been studied so far. We believe that conversion from responding to medical treatment to a refractory course may be more important than the duration of the disease. A better outcome is expected in patients who can still achieve a high PLT level responding to medical treatment and do not present adverse effects of medications, which may be optimal timing for LS.

Another interesting aspect of this study was finding that accessory spleens were present in 21.82% patients in general, and this did not differ between groups. This seems not to be a significant clinical finding, yet there were some case reports about recurrent ITP [28,29].

The present study is limited by typical factors for a single-center retrospective analysis. It has a retrospective design. Due to a long period of study duration (19 years) we were not able to precisely analyze hematological preparation for surgery and preoperative conservative treatment. However, the study analyzes remission rates after splenectomy, not preoperative treatment. Indications have not changed over that time, with only patients unsuitable for further conservative treatment operated on. Follow-up is different mostly due to the time interval between the date of surgery and our analysis (follow-up). To collect long-term data, we contacted the patients and checked their remission status. The minimal time interval between surgery and follow-up was one year after surgery. Additionally, some patients were lost to follow up and we did not include them in the analysis. However, the study group is still relatively large and permitted the drawing of reliable results and conclusions. Moreover, patients were operated on by several surgeons with different levels of experience. Another limitation was that we did not include potential postoperative complications that might have occurred later than 30 days after discharge in this analysis. Those complications may have influenced the overall complication rate.

## 5. Conclusions

Laparoscopic splenectomy is a safe and effective treatment of pharmacologically refractory ITP. The majority of patients achieved long-term responses in this study. Independent prognostic factors for ITP remission after LS revealed in our study are: age <41 years, BMI < 2 4.3 kg/m^2^, and preoperative platelet count ≥97 × 10^3^/mm^3^. Duration of ITP and treatment time were unrelated to remission after LS. Further studies are necessary to assess patient-related predictive factors of response to surgical therapy for determination of optimal approach and optimal surgical timing.

## Figures and Tables

**Table 1:** Patients’ characteristics.

	All *n* = 165 (100%)	Group 1 *n* = 113 (68.48%)	Group 2 *n* = 52 (31.52%)	*p*-Value
Gender, *n* (%) male female	52 (32) 113 (68)	34 (30) 79 (70)	18 (35) 34 (65)	0.561
Age, median (IQR), years	35 (25–52)	31 (24–48)	50 (36.5–60)	**<0.001**
BMI, median (IQR), kg/m^2^	25.69 (21.76–29.30)	24.30 (20.52–29.01)	27.65 (25.50–30.62)	**<0.001**
Spleen size, median (IQR), cm	11 (10–12)	11 (10–12)	11 (10–12)	0.599
Lowest preoperative platelet count, median (IQR), ×10^3^/mm^3^	8 (4–16)	10 (5–18)	7 (3–12)	0.071
Preoperative platelet count, median (IQR), ×10^3^/mm^3^	90 (48–119)	97 (50–125)	68.5 (36.5–107)	**0.034**
Preoperative steroids administration, *n* (%)	158 (95.76)	107 (94.69)	51 (98.08)	0.293
Immunoglobulin administration, *n* (%)	37 (22.42)	26 (23.01)	11 (21.15)	0.791
Preoperative platelet transfusions, *n* (%)	19 (11.52)	9 (7.96)	10 (19.23)	0.065
Accessory spleen, *n* (%)	36 (21.82)	25 (22.12)	11 (21.15)	0.950
Perioperative complications, *n* (%)	13 (7.88)	9 (7.96)	4 (7.69)	0.610
Blood transfusions, *n* (%)	3 (1.82)	1 (0.88)	2 (2.85)	0.234
Time from diagnosis of ITP to procedures, median (IQR), months	24 (6.75–57)	18 (6.5–48)	24 (9–84)	0.241
Symptomatic ITP, *n* (%)	90 (54.55)	59 (52.21)	31 (59.62)	0.375
Preoperative time of conservative treatment, median (IQR), months	9.5 (5–30)	8.5 (4.5–24)	12 (5–51)	0.178

**Table 2:** Perioperative morbidity.

		All	Group 1	Group 2
Operative time, median (IQR), min		85 (65–105)	80 (60–100)	90 (70–110)
Blood loss, median (IQR), mL		50 (20–100)	30 (10–50)	50 (20–100)
LOS, median (IQR), days		4 (3–4)	3 (3–4)	4 (3–5)
Perioperative morbidity, *n* (%)		13 (7.88)	9 (7.96)	4 (7.69)
Clavien–Dindo	Morbidity	All	Remission	Non-remission
**IIIb**	Acute pancreatitis, sub-phrenic abscess	1	1	0
	Gastric perforation, sub-phrenic abscesses	1	1	0
	Peritonitis, intra-abdominal abscesses	1	1	0
	Intra-abdominal bleeding	5	2	3
**IIIa**	Pancreatitis	1	0	1
**II**	Pneumonia	2	2	0
	Postoperative fever	1	1	0
**I**	Sub-phrenic fluid collection	1	1	0

**Table 3:** Univariate logistic regression model for factors potentially affecting remission of immune thrombocytopenic purpura (ITP) after laparoscopic splenectomy (LS).

	OR	95% CI	*p*-Value
Male/Female	1.23	0.61–2.49	0.561
Age, with every 1 year	0.94	0.93–0.97	**<0.001**
BMI, with every 1 kg/m^2^	0.85	0.77–0.93	**<0.001**
LOS, with every 1 day	0.82	0.59–1.16	0.263
Ultrasound length of spleen, with every cm	1.04	0.93–1.16	0.522
Lowest preoperative platelet count, with every 1 × 10^3^/mm^3^	1.03	0.99–1.07	0.080
Preoperative platelet count, with every 1 × 10^3^/mm^3^	1.01	1.00–1.01	**0.025**
Preoperative steroids administration	0.35	0.04–3.03	0.337
Preoperative immunoglobulin administration	1.11	0.50–2.49	0.791
Platelet transfusions	0.36	0.14–0.97	**0.041**
Additional spleen	1.06	0.47–2.37	0.889
Perioperative complications	1.03	0.30–3.56	0.952
Blood transfusions	0.22	0.02–2.57	0.223
Operative time, with every 1 min	0.99	0.98–1.01	0.671
Blood loss, with every 1 mL	0.99	0.98–1.01	0.696
Time from diagnosis of ITP to procedures, with every 1 month	0.99	0.99–1.00	0.396
Symptoms	0.74	0.38–1.45	0.376
Preoperative time of conservative treatment, with every 1 month	0.99	0.98–1.02	0.230

**Table 4:** Multivariate logistic regression model for factors potentially affecting remission of ITP after LS.

	OR	95% CI	*p*-Value
Age <41 years	4.49	1.66–12.09	**0.003**
BMI < 24.3 kg/m^2^	4.67	1.44–15.16	**0.010**
Preoperative platelet count ≥97 × 10^3^/mm^3^	3.50	1.30–9.47	**0.012**
Platelet transfusions	0.75	0.21–2.75	0.665

**Table 5:** Univariate logistic regression model for factors potentially affecting postoperative platelet count >50 × 10^3^/mm^3^.

	OR	95% CI	*p*-Value
Male/Female	1.44	0.49–4.23	0.505
Age, with every 1 year	0.95	0.92–0.98	**0.001**
BMI, with every 1 kg/m^2^	0.92	0.82–1.03	0.131
LOS, with every 1 day	1.02	0.73–1.45	0.889
Ultrasound length of spleen, with every cm	1.15	0.90–1.47	0.254
Lowest preoperative platelet count, with every 1 × 10^3^/mm^3^	1.02	0.97–1.07	0.441
Preoperative platelet count, with every 1 × 10^3^/mm^3^	1.03	1.00–1.06	**0.010**
Preoperative steroids administration	0.51	0.20–1.32	0.162
Preoperative immunoglobulin administration	0.63	0.22–1.80	0.635
Platelet transfusions	0.43	0.27–0.71	**0.048**
Additional spleen	1.67	0.46–6.11	0.435
Perioperative complications	2.43	0.01–4.65	0.981
Blood transfusions	0.37	0.67–1.69	0.695
Operative time, with every 1 min	0.99	0.97–1.03	0.113
Blood loss, with every 1 mL	1.00	0.99–1.04	0.607
Time from diagnosis of ITP to procedures, with every 1 month	1.00	0.99–1.06	0.945
Symptoms	0.98	0.38–2.55	0.966
Preoperative time of conservative treatment, with every 1 month	0.99	0.98–1.02	0.248

**Table 6:** Multivariate logistic regression model for factors potentially affecting postoperative PLT > 50 × 10^3^/mm^3^.

	OR	95% CI	*p*-Value
Age <42 years	4.93	1.49–16.34	**0.009**
Preoperative platelet count ≥95 × 10^3^/mm^3^	3.45	1.03–11.59	**0.043**
Platelet transfusions	0.69	0.23–2.07	0.501
